# Complete Genome Sequence of the Marine Bacterium Erythrobacter flavus Strain KJ5

**DOI:** 10.1128/MRA.00140-19

**Published:** 2019-03-28

**Authors:** Yu Kanesaki, Edi Setiyono, Delianis Pringgenies, Ryota Moriuchi, Tatas H. P. Brotosudarmo, Koichiro Awai

**Affiliations:** a Research Institute of Green Science and Technology, Shizuoka University, Shizuoka, Japan; b Ma Chung Research Center for Photosynthetic Pigments, Universitas Ma Chung, Malang, Indonesia; c Faculty of Fisheries and Marine Science, Diponegoro University, Semarang, Indonesia; d Department of Biological Science, Faculty of Science, Shizuoka University, Shizuoka, Japan; e Research Institute of Electronics, Shizuoka University, Shizuoka, Japan; University of Southern California

## Abstract

Erythrobacter flavus strain KJ5 (formerly called *Erythrobacter* sp. strain KJ5) is a yellowish marine bacterium that was isolated from a hard coral in the Karimunjawa Islands of Indonesia. Here, we report the complete genome sequence of the bacterium and provide a useful resource for studies of the biosynthetic pathways of its unique carotenoids.

## Abstract (precis)

Erythrobacter flavus strain KJ5 (formerly called *Erythrobacter* sp. strain KJ5) is a yellowish marine bacterium that was isolated from a hard coral in the Karimunjawa Islands of Indonesia.

## ANNOUNCEMENT

Erythrobacter flavus strain KJ5 (formerly called *Erythrobacter* sp. strain KJ5) is a yellowish marine bacterium that was isolated from a tropical sea of the Karimunjawa Islands in Indonesia (1). This bacterium was originally found as a possible symbiont of Acropora nasuta, a hard coral, and was found to be close relative of Erythrobacter flavus strain SW-46 according to its 16S RNA sequence (1). Preliminary analysis of the pigment content of this bacterium revealed that it lacks bacteriochlorophyll *a* but possesses at least 16 species of carotenoids, including β-carotene and zeaxanthin (2). Molecular species of the three most dominant carotenoids are still unknown, but they have been found to have more polarity than zeaxanthin (2). Moreover, two of these three carotenoids are diminished by saponification, indicating that these two pigments might be esterified by hydrophilic compounds (e.g., sugars, sulfate) (2). We analyzed the exact structures of these carotenoids, and simultaneously, we performed genome sequence analysis of strain KJ5 to determine the biosynthetic pathways of these carotenoids.

Strain KJ5 was cultivated in an *Erythrobacter* medium as reported by Shioi (3). Genomic DNA was extracted using the DNeasy blood and tissue kit (Qiagen, Venlo, Netherlands) with the manufacturer’s instructions. The sequencing library was synthesized using the TruSeq DNA Nano kit (Illumina, Inc., San Diego, CA). Whole-genome sequencing was performed by a massive parallel MiSeq sequencer (Illumina, Inc.) with a read length of 302 bp and an average insert size of 800 bp. Reads were filtered for quality values of >30 with the adaptor trimming option using the CLC Genomics Workbench v.10.0.1. (Qiagen). *De novo* genome assembly was performed using SPAdes v.3.0.1 (4) and was checked with QUAST v.5.0.2 (5) and Bandage v.0.8.1 (6), both with default settings. The single assembly was finalized by removing the terminal overhanging region. Gene prediction and functional annotation were performed using the DFAST pipeline v.1.1.4 with default settings (7). The start codon of the *dnaA* gene was defined as the +1 position of the chromosome. The mean genome coverage was 103×.

The complete genome sequence of strain KJ5 consists of a 2,819,202-bp circular chromosome with an average G+C content of 63.92%. Functional annotation revealed a total of 2,713 coding sequences, 48 tRNA genes, and 2 copies of the rRNA operons. The 16S rRNA gene sequence of strain KJ5 has 99.89% similarity with that of Erythrobacter flavus strain VG1, isolated from the Red Sea (GenBank accession number NZ_CP022528), according to a BLASTn search using the 16S rRNA gene sequence of strain KJ5 as a query and using strain VG1 as a reference. The synteny of the genes was also highly similar between strain KJ5 and strain VG1 over the whole genomic sequences, which were analyzed by constructing a genome rearrangement map using amino acid sequences by Genome Traveler v.3.0.25 with default settings (In Silico Biology, Inc., Yokohama, Japan).

According to the annotation, strain KJ5 was found to lack the genes encoding enzymes responsible for biosynthesis from protoporphyrin IX to bacteriochlorophyll *a*. On the other hand, for the carotenoid synthetic pathway, all the genes responsible for biosynthesis from geranylgeranyl diphosphate to β-carotene and zeaxanthin were found. These results completely fit the pigment content of the bacterium (2).

### Data availability.

The genome sequence of Erythrobacter flavus strain KJ5 has been deposited in DDBJ/ENA/GenBank under the accession number AP019389. The accession number of the original read data set in the SRA is DRR162742.
